# Individual and community level associates of contraceptive use in Ethiopia: a multilevel mixed effects analysis

**DOI:** 10.1186/s13690-019-0371-z

**Published:** 2019-10-30

**Authors:** Masrie Getnet Abate, Amare Abera Tareke

**Affiliations:** 1 Biostatistics Unit, Department of Epidemiology, Institute of Health, Jimma University, Jimma, Ethiopia; 2 Department of Biomedical Sciences, Institute of Health, Jimma University, Jimma, Ethiopia

**Keywords:** Contraceptive use, Ethiopia, Individual and community-level characteristics, Multilevel analysis

## Abstract

**Background:**

Family planning is one of the four pillars of safe motherhood initiative to reduce maternal death in developing countries. Despite progress in contraceptive use, unmet needs are wide open and fertility remains high. Ethiopia have a higher fertility rate which contributes to maternal and child health destitution, putting pressure on the already weak health system. This study examined individual and community-level factors associated with contraceptive use in Ethiopia.

**Methods:**

Data from Ethiopian Demographic and Health Survey 2016 were used to identify individual and community level associated factors among reproductive-age women. Non-pregnant, fecund and sexually active women aged 15–49 were included. Six hundred forty-two communities and 6854 women were involved from this two-stage cluster sampled data. The analysis was done using two-level mixed-effects logistic regression to determine fixed effects of individual and community-level factors and random intercept of between characteristics.

**Results:**

From the total eligible women for contraceptive use 2393 (34.9%) of them were users. Injectables were the commonest of all contraceptive methods. Various individual-level variables were associated with contraceptive use. Household wealth index, women’s age, number of living children, husband’s occupation, ever experience of a terminated pregnancy, current working status of the women, number of births in the last 3 years, and hearing of FP messages through different media were significantly associated individual-level variables after adjusting other factors. Community characteristics like region, place of residence, religion, and community-level wealth were the factors associated with contraceptive use.

**Conclusion:**

Both individual and community-level characteristics were significant predictors of use of contraceptives in Ethiopian women. Besides the individual-level factors, interventions should also consider community-level associates.

## Background

In the current growth rate of the world population, 1.13%, about 80 million people will be added per year [1]. The United Nations estimates the World population will increase from 7.4 billion in 2016 to 8.1 billion in 2025, with the most growth in developing countries and more than half in Africa [2]. With the highest rate of population growth, Africa is expected to account for more than half of the world population growth between 2015 and 2050 [2]. Ethiopia, a second populous nation in Africa, had an average of 4.6 children per women in 2016 [3]. This figure puts Ethiopia among countries with higher fertility rates in the world. Fertility rates are determinant factors in human development index (HDI) affecting life expectancy, education, per capita and other indicators [4]. The role of modern contraceptives in decreasing maternal and child mortality, and other health costs thereby improving maternal and child health is widely advocated [5, 6].

The use of modern contraceptives ranges from 3.6% in the Somali region to 56.3% in Addis Ababa with a national prevalence of 27.3% in 2011 [7]. The use of modern contraceptives has variation with different factors, creating difficulty to determine the national figure and its determinants. Although there are recent improvements, developing countries including Ethiopia, carry the most significant proportion of maternal and child mortality with multi-factorial etiology [8, 9]. The major obstacle for improvements in maternal and child health services is continued population growth with sustained high fertility rate, placing considerable strain on the already fragile health system [9]. Contraceptive methods are currently recommended to decrease higher total fertility rates especially in developing countries like Ethiopia [10].

Modern contraceptives in Ethiopia are available in every health facility free of cost and pushing to increase utilization. The use of contraceptives in Ethiopia usually focused on short-acting methods like birth control pills and injectable, which have a high chance of discontinuation [11, 12]. Fears of side effects, health concerns, low knowledge of women, lower educational level, men’s (partner’s) objection, male partner desire for more children, and lack of women’s decision making power are barriers for contraceptive utilization especially in long-acting and permanent methods [13].

The number of children, women’s current age, age at first marriage, education, religious affiliation, media exposure about family planning, wealth index, occupation, husband’s occupation, place of residence, husbands approval couple’s discussion, are the main determinant factors for utilization of modern contraceptives among married women of reproductive age [11, 12, 14, 15].

Several community-level factors were found to be associated with contraceptive use elsewhere. Access to family panning in nearby store or health facility, female autonomy, female education, religion, place of residence, proportions of polygynous marriages and community level media exposure were community-level determinants in Nigeria [16, 17]. Average community wealth and the women’s relative socioeconomic position within the community have significant positive effects on the use of modern contraceptives in Mozambique. And also, the contextual effects due to community wealth were greater in rural than in urban areas [18]. A study in Poland found that the contraceptive behavior of friends and family is more influential than are women’s own characteristics and that community-level characteristics additionally influence contraceptive use [19].

Representative studies on the use and determinants of modern contraceptives in Ethiopia are scares. Besides deficiency of fertile evidence, small inconclusive studies in different areas without considering community-level factors put different dissimilar, mutually exclusive determinant factors, lacking to draw relevant conclusions in national level.

Generally, even if previous studies have given important clues to policymakers, programmers, and other stakeholders, they mainly lack consistency and representatives to be used for national-level policy making and programming by concerned bodies. Thus, the present study aims to determine multilevel determinants of modern contraceptive use among married women of the reproductive age group in Ethiopia using the 2016 Ethiopian Demographic Health Survey (EDHS 2016) data.

## Methods

### Study design and setting

Ethiopia is the second populous country in Africa next to Nigeria with a population of more than one hundred million. Administratively, Ethiopia is divided into nine geographical regions (Tigray, Afar, Amhara, Oromia, Somali, Benishangul-Gumuz, SNNPR, Gambella and Harari) and two administrative cities, Addis Ababa and Diredawa. Ethiopia shares the boundaries in the North with Eritrea, in the South with Kenya and Somalia, in the West with South Sudan and North Sudan, in the East with Djibouti and Somalia. The survey was a population-based cross-sectional study conducted from January 18, 2016, to June 27, 2016, across the country [20].

### Data source

The dataset used in this study was obtained from MEASURE DHS database at http://dhsprogram.com/data/ after getting the approval letter from DHS program office for 2016 Ethiopian Demographic and Health Survey, which is the fourth comprehensive survey.

### Sampling procedures

The 2016 EDHS sample was stratified and selected in two stages. In the first stage, a total of 645 clusters (202 in urban areas and 443 in rural areas) were selected based on 2007 population housing census. A household listing was carried out in all of the selected clusters from September to December 2015, served as a sampling frame for the selection of households in the second stage.

**Fig. 1:**
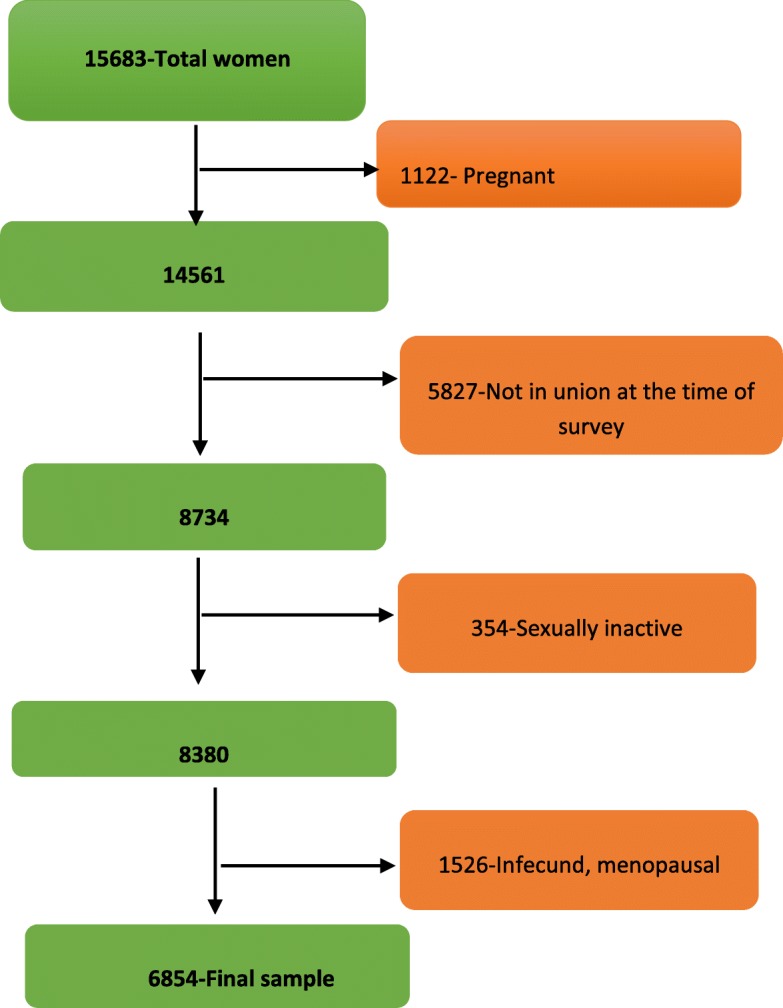
Schematic illustration of women included in the study. From women’s health survey 15,683 women participated in the survey. Several exclusion criteria were used to reach the final sample for this study. Pregnant women were 1122 during the survey period and were excluded in the first step. The other criteria used for eligibility to this study was women should be married or were in union with their couple during the survey. A significant number of women excluded by failing to fulfill this criterion. After screening for sexual activity and fecundity we reached the final sample size of 6854

A representative sample of 18,008 households was selected for the 2016 EDHS. From the interviewed households, 16,583 eligible women were identified for individual interviews. Interviews were completed with 15,683 women, yielding a response rate of 95% [16]. In this study, a total of 6854 eligible women from 642 clusters (201 in urban and 441 in rural) were included after the necessary exclusion criteria were carried out, summarized as Fig. 1.

### Study variables

#### Dependent variable

The dependent variable is current contraceptive use among married women in reproductive age. Current use of contraceptives was dichotomized, women were categorized as “users” to indicate the use of any modern contraceptive methods and “non-users” to represent those who were not using any contraceptive method or using Folkloric and Traditional methods.

#### Independent variables

The independent variables for contraceptive utilization were based on the previous literature and availability of the variable on 2016 EDHS dataset. Variables were broadly classified into two main groups, individual level and community level variables aligned for multilevel analytic approach.

#### Individual-level variables

Age of women at the time of survey, educational level of women, household wealth index, total number of living children, husband’s occupation, exposure to mass media, experience of terminated pregnancy, history of smoking cigarettes, current working status of women, births in the last 3 years, hearing of family planning messages through media, Visit of health facility in the last 12 months and husband’s desire for children were included as individual-level variables.

#### Community-level variables

Region and place of residence were regarded as community-level variables directly from EDHS but the remaining variables are not available directly from EDHS. Hence, we aggregated the variables at the community-level based on the individual information and then the aggregated values were classified as low and high if the median values or the proportions of the clusters were below and above the national level respectively. Having this, community-level religious affiliation, community-level media exposure about family planning, community level women employment, community-level of health facility visit and community-level wealth were considered as community-level variables.

#### Region

The EDHS was collected from administratively divided of nine regions and two federal’s administrative cities. We used these administrative boundaries as community-level factor.

#### Place of residence

It was classified as rural and urban, considered as a community level factor.

#### Community-level religion

From the distribution of Muslims in the cluster, the proportion of Muslims was taken and a cut of value of 42.7% was considered. Clusters having greater than 42.7% of women Muslims regarded as high community level Muslim prevalence and less than 42.7% of women Muslims in the cluster was considered as low community level Muslim prevalence.

#### Community-level media exposure

Information heard about family planning from radio, TV, and magazine/newspaper were considered together. If a woman had exposure for at least one of the three, we consider as she had media exposure. If greater than or equal to 50% of the cluster members heard information, we considered as having high community-level information. If less than 50% of the cluster members had no information, the cluster was classified as having low information from media.

#### Community-level employment

Classified based on national women employment proportion. Thirty-three percent of women nationally were working during the survey. Clusters were classified high community-level employment if greater than or equal to 33% of the members were currently working, or low if less than 33% of cluster members were not working.

#### Community-level visit of health facility

Classified as high and low by taking 51% cluster members as the reference according to the national data.

#### Community-level wealth

Classified based on the wealth index of the household. The median value of the wealth index at the national level was 3. Then the aggregated clusters were classified into low if the median value of the cluster was below 3 and high if the median value of the cluster was greater than or equal to 3.

### Data analysis

In this study, two-level mixed-effects logistic regression analyses were employed using R software (version 3.5.3). Since the EDHS data was hierarchical, i.e., women were nested in household and household were nested in cluster multi-level analysis is recommended. First, bivariate two-level mixed-effect logistic regression analyses were done to assess the association between the independent variables and the dependent variable of the study. The overall categorical variables with a *p*-value of < 0.25 at the bivariate two-level mixed-effect logistic regression analysis were included into the final model of multivariable two-level mixed effect logistic regression model in which odds ratio with 95% confidence intervals were estimated to identify independent variables of modern contraceptive use. *P* values less than 0.05 were employed to declare statistical significance. Fixed effect and random effect were calculated to assess the individual and cluster variations respectively. Moreover, the descriptive statistics were displayed for the community and individual variables.

Thus, four models are displayed in this analysis, Null model (model containing no factors), Model I (containing only individual factors), Model II (containing only community factors) and Model III (both individual and community-level factors). The fitted model was:

$$ \mathit{\log}\left[\frac{\pi_{ij}}{1-{\pi}_{ij}}\right]={\beta}_0+{\beta}_1{\mathrm{X}}_{1 ij}+\dots {\beta}_n{\mathrm{X}}_{nij}+{uo}_j+{e}_{ij}, $$

Where

✓ *π*_*ij* _-the probability of women who currently using contraceptive

✓ 1 − *π*_*ij*_ - the probability of not using modern contraception

✓ *β*_0_ is log odds of the intercept

✓ *β*_1_…. *β*_*n*_ - the amount of effect by the individual and community-level variables

✓ Χ_1_…Χ_*n*_ - the independent variables at individual and community level

✓ *uo*_*j*_ - the random error at community(cluster) and

✓ *e*_*ij*_ - the random error at the individual level.

The intra-class correlation (ICC) was calculated as the proportion of the between cluster variation in the total variation:

$$ ICC=\frac{Var\left({u}_{oj}\right)}{Var\left({uo}_j\right)+\raisebox{1ex}{${\pi}^2$}\!\left/ \!\raisebox{-1ex}{$3$}\right.}, $$

Where,

✓ *Var*(*u*_*oj*_) is the community (cluster) level variance.

✓ $$ \raisebox{1ex}{${\pi}^2$}\!\left/ \!\raisebox{-1ex}{$3$}\right. $$ is the standard logistic distribution, that is, the assumed household variance component, which is $$ \raisebox{1ex}{${\pi}^2$}\!\left/ \!\raisebox{-1ex}{$3$}\right.\approx $$ 3.29.

The variability on the odds of modern contraceptive users explained by successive models were calculated by Proportional Change in Variance (PCV) as:

$$ PCV=\frac{V_e-{V}_{mi}}{Ve}, $$

Where,

✓ *V*_*e*_ - the variance in modern contraceptive utilizations in the null model.

✓ *V*_*mi*_ - the variances in the successive models.

## Results

### Prevalence of contraceptive use

**Fig. 2:**
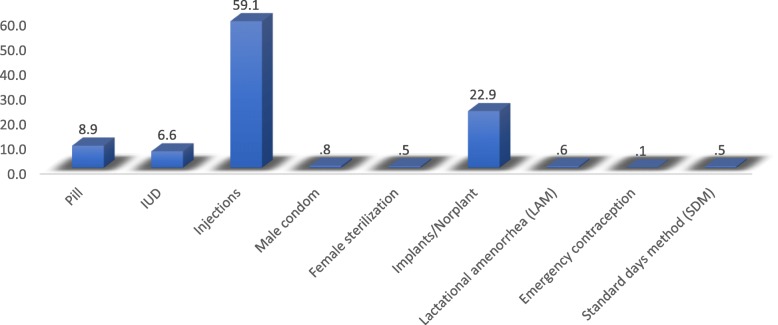
Percentage of type of contraceptive methods among users in Ethiopia, 2016 Ethiopian Demographic and Health Survey. As depicted in Fig. 2, closer to three-fourth of contraceptive users in Ethiopia uses either injections or Norplant’s. Almost six from ten users use injections as a contraceptive method. Norplant was used by 22.9% of users and ranked second to injections. Pills and IUD contributed to 15% of the total percentage, and other contraceptive methods contributed less than 3%

From 2016 EDHS data set a total of 6854 married, non-pregnant and fecund women were included for this study. Of these fecund women, 2393 (34.9%) of them were users of contraceptive methods. Injectables were the most commonly used contraceptive methods (59.1%), the percentage of users in each contraceptive type is summarized in Fig. 2.

More than half of the women had no formal education [3891(56.8%)], and only 14.2% of them had attended secondary education or higher. By contrast, their husband had a better education with a decreased number of no formal education males 2981(43.5%), again 28.3% of them had secondary education or higher. More than two-thirds of women 4696 (68.5%) had either one or two under-five children, while less the one-fourth of the total 1446(21.1) had no under-five children. Majority of the women 4674(68.2%) were not working in the time of the survey, this figure is bigger when we consider the husband’s status of 681(9.9%), indicating disparities in work. Summarized in Table 1: Background characteristics of married non-pregnant women, Ethiopian Demographic and Health Survey 2016 (*n* = 6854).

**Table 1:** Background characteristics of married non-pregnant women, Ethiopian Demographic and Health Survey 2016 (*n* = 6854)

Individual Variables	Categories	Frequency	%
Age in 5-year groups	15–19	500	7.3
	20–24	1343	19.6
	25–29	1668	24.3
	30–34	1357	19.8
	35–39	1120	16.3
	40–44	607	8.9
	45–49	259	3.8
Highest educational level			
	No education	3891	56.8
	Primary	1922	28.0
	Secondary	644	9.4
	Higher	397	5.8
Religion			
	Christians	3840	56.0
	Muslin	2927	42.7
	Other	87	1.3
Number of children 5 and under in household			
	None	1446	21.1
	1–2	4696	68.5
	> = 3	712	10.4
Number of living children categories			
	None	665	9.7
	1–4	4154	60.6
	5–8	1879	27.4
	> = 9	156	2.3
Births in last 3 years category			
	None	2539	37.0
	one birth	3669	53.5
	more than two births	646	9.4
Wealth index combined			
	Poor	3077	44.9
	Middle	971	14.2
	Rich	2806	40.9
Respondent currently working			
	No	4674	68.2
	Yes	2180	31.8
Exposure to mass media			
	No	4045	59.0
	Yes	2809	41.0
History of terminated pregnancy			
	No	6165	89.9
	Yes	689	10.1
Heard family planning through media			
	No	4764	69.5
	Yes	2090	30.5
Visited by fieldworker in last 12 months			
	No	4837	70.6
	Yes	2017	29.4
Did fieldworker talk about family planning			
	No	787	36.5
	Yes	1230	63.5
Visited health facility in the last 12 months			
	No	3322	48.5
	Yes	3532	51.5
At health facility, told of family planning			
	No	2156	61.0
	Yes	1376	39.0
History of smoking Cigarette			
	No	6786	99.0
	Yes	68	1.0
Covered by health insurance			
	No	6572	95.9
	Yes	282	4.1
Husband’s desire for children			
	Both want same	2781	40.6
	Husband wants more	1775	25.9
	Husband wants fewer	465	6.8
	Don’t know	1820	26.6
Husband/partner’s education level			
	No education	3045	44.4
	Primary	2213	32.3
	Secondary	868	12.7
	Higher	728	10.6
Husband/partner’s occupation (grouped)			
	Did not work	681	9.9
	Clerical/sales/services/skilled labor	1572	22.9
	Professional/technical/managerial	4601	67.1
Person who usually decides on respondent’s health care			
	Respondent alone	1242	18.1
	Respondent and husband/partner	4343	63.4
	Husband/partner alone	1234	18.0
	Someone else	27	0.4
	Other	8	0.1
Current use of contraceptive methods			
	No	4461	65.1
	Yes	2393	34.9

Exposure to mass media including radio, television, magazine/newspaper, at least for one of the media, remains below half of the study population. Only 41% of the women had exposure to medias 12 months preceding the survey. Media exposure for family planning was also low with 2090(30.5%) of mothers heard through radio or TV or newspaper in the last few months before the survey. Health insurance coverage was extremely low as well. More than 95% of the women were no insured. While 1 in 10 mothers experienced a terminated pregnancy, smoking in reproductive age fecund Ethiopian women was low (1%). The mean age of mothers during the survey was 29 years, the median value for under-five children in the household during the survey was one child and the mean value for the number of total living children in the household preceding to the 2016 EDHS were three.

From the total 645 EDHS clusters, 642 clusters had married, non-pregnant reproductive age women eligible for contraceptive use and included in this study. Below one-third of clusters were from urban areas. Three hundred eighty-two (59.5%) of clusters had lower Muslim proportions at the community level. Comparing with the median educational level of the country, women in 306 (55.8%) clusters had lower education. Almost half of the clusters had lower employment status, and media exposure about family planning was poor even at community-level. Description of community-level variables was presented in Table 2 Description of cluster background characterstics among reproductive age women in Ethiopia, Ethiopian Demographic and Health Survey 2016 (*n* = 642).

**Table 2:** Description of cluster background characterstics among reproductive age women in Ethiopia, Ethiopian Demographic and Health Survey 2016 (*n* = 642)

Community level variables	Categories	Frequency	(%)
Region			
	Tigray	722	10.5
	Afar	589	8.6
	Amhara	839	12.2
	Oromia	933	13.6
	Somali	618	9.0
	Benishangul	569	8.3
	SNNPR	817	11.9
	Gambela	439	7.1
	Harari	380	5.5
	Dire dawa	406	5.9
	Addis Ababa	492	7.2
Type of place of residence			
	Urban	201	31.3
	Rural	441	68.7
Muslim Religion at the community level			
	low	382	59.5
	high	260	40.5
Wealth			
	Low	269	41.9
	High	373	58.1
Employment status of women at the community level			
	low	317	49.4
	high	325	50.6
Heard family planning on media at community level			
	low	363	56.5
	high	279	43.5
Media exposure at community level			
	low	253	39.4
	high	389	60.6
Husbands desire for more children			
	Low	283	44.1
	High	359	55.9
Terminated pregnancy			
	low	369	57.5
	high	273	42.5
Visit health institution			
	Low	257	40.0
	High	385	60.0
Visited by field worker at the community level			
	low	361	56.2
	high	281	43.8
Coverd by health insurance			
	Low	525	81.8
	High	117	18.2

### Multilevel analysis

A two-level mixed-effect logistic regression was used to analyze the effect of women’s individual characteristics and community-level factors in determining women’s use of contraceptives. As depicted in the empty model 40% of the total variance in the odds of contraceptive use was accounted by between cluster variation of characteristics. The between cluster variability declined over successive models from 40% in the empty model into 23% in individual-level only model, 16% in community-level factors only model and 14% in the combined model. Thus, the combined model of individual-level and community-level factors were preferred for predicting women’s contraceptive use.

### Individual level determinants

In the model I only individual-level variables were added. The result signified that wealth index, women’s age, number of living children, husband’s occupation, ever experience of a terminated pregnancy, current working status of the women, number of births in the last 3 years, and hearing of family planning messages through different media were significantly associated variables with contraceptive use.

The ICC in Model I indicated that 23% of the variation in contraceptive use was attributable to difference across the communities. From PCV in Tables 3, 82.5% of the variance in contraceptive use across communities was explained by individual-level factors.

**Table 3:** Multilevel regression results assessing effects of individual and community level characteristics on contraceptive use among women in Ethiopia, 2016 Ethiopian Demographic and Health Survey

Variables/ characteristics	Null model	Model I	Model II	Model III
		Individual characteristics	Community level characteristics	Individual and community-level characteristics
		OR (95%CI)	OR (95%CI)	OR (95%CI)
Educational level of women				
No formal education ^(Ref)^				
Primary		1.3(1.1, 1.5) **		1.2(1.0, 1.4)
Secondary		1.4(1.1, 1.8) **		1.2(0.9, 1.5)
Higher		1.5(1.1, 2.0) **		1.2(0.8, 1.6)
Wealth index				
Poor^(ref)^				
Middle		1.9(1.5, 2.3) ***		1.4(1.1, 1.7) ***
Rich		2.2(1.8, 2.6) ***		1.4(1.1, 1.7) ***
Age				
35–39^(ref)^				
15–19		1.6(1.1, 2.2) **		2.1(1.5, 2.9) ***
20–24		2.1(1.6, 2.7) ***		2.5(2.0, 3.2) ***
25–29		1.7(1.3, 2.1) ***		1.8(1.5, 2.3) ***
30–34		1.3((1.0, 1.6) *		1.4(1.1, 1.7) **
40–44		1.0(0.8, 1.4)		1.0(0.8, 1.3)
45–49		0.6(0.4, 0.9) *		0.6(0.4, 0.8) **
Number of living children				
None^(ref)^				
1–4		3.6(2.7, 4.7) ***		3.6(2.8, 4.7) ***
5–8		3.5(2.5,4.9) ***		4.0(2.8, 5.5) ***
> =9		2.5(1.3, 4.8) **		3.2(1.7, 6.1) ***
Husband/partner’s occupation				
Did not work^(ref)^				
Farming/unskilled		2.0(1.5, 2.6) ***		1.6(1.3, 2.1) ***
Clerical/sales/services/skilled labor		2.1(1.6, 2.8) ***		1.9(1.4, 2.5) ***
professional/technical/manager		1.9(1.4, 2.7) ***		1.9(1.4, 2.7) ***
Others		1.5(1.0, 2.2) *		1.4(0.9, 2.0)
Exposure to mass media				
No^(ref)^				
Yes		1.2(1.0, 2.2) *		1.1(1.0, 1.4)
Ever had a terminated pregnancy				
No^(ref)^				
Yes		0.7(0.6, 0.9) *		0.7(0.6,0.9) **
Smokes cigarettes				
No^(ref)^				
Yes		0.8(0.4, 1.5)		0.8(0.4, 1.6)
Respondent currently working status				
No^(ref)^				
Yes		1.3(1.1, 1.4) ***		1.3(1.1, 1.5) **
Births in the last 3 years				
No birth^(ref)^				
One birth		0.5(0.4, 0.6) ***		0.5(0.4, 0.6) ***
Two and more births		0.2(0.1, 0.3) ***		0.3(0.2, 0.4) ***
Heard FP messages through media at least once				
No^(ref)^				
Yes		1.2(1.0, 1.5) *		1.2(1.0, 1.4)
Visited Health Facility in the last 12 months				
No^(ref)^				
Yes		1.4(1.3, 1.6) ***		1.3(1.1, 1.5) ***
Husbands desire for children				
Both wants the same^(ref)^				
Husband wants more		0.8(0.7,0.9) **		0.8(0.7, 1.0) *
Husband wants fewer		1.0(0.7, 1.2)		0.9(0.7, 1.2)
Don’t know		0.8(0.7,0.9) **		0.8(0.7, 0.9) **
Region				
Tigray			1.3(0.8, 2.0)	1.3(0.9, 2.0)
Afar			0.3(0.2, 0.6) ***	0.4(0.2, 0.6) ***
Amhara			2.6(1.7, 4.0) ***	2.7(1.8, 4.1) ***
Oromia			1.5(0.9,2.3)	1.5(1.0, 2.3)
Somali			0.1(0.04, 0.2) ***	.1(0.05, 0.2) ***
Benishangul			1.3(0.8, 2.1)	1.3(0.8, 2.0)
South Nations Nationalities and Peoples’ Region			2.0(1.3, 3.1) **	2.1(1.4, 3.3) ***
Gambela			0.8(0.5, 1.3)	0.8(0.5, 1.3)
Harari			1.0(0.6, 1.6)	0.9(0.6, 1.4)
Dire dawa			0.8(0.5, 1.3)	0.8(0.5, 1.3)
Addis Ababa^(ref)^				
Place of residence				
Urban^(ref)^				
Rural			0.5(0.4, 0.7) ***	0.7(0.5,0.9) *
Community-level women employment				
low^(ref)^				
High			1.3(1.0, 1.5) *	1.1(0.9, 1.3)
Wealth index				
low^(ref)^				
High			2.3(1.8, 2.9) ***	1.7(1.4, 2.1) ***
Media exposure				
low^(ref)^				
High			1.4(1.1, 1.8) **	1.2(0.9, 1.5)
Proportion of Muslims in the cluster				
low^(ref)^				
High			0.6(0.5, 0.8) ***	0.6(0.5, 0.8) ***
Health facility visit				
Low^(ref)^				
High			1.3(1.0, 1.6) *	1.1(0.9,1.4)

*P < 0.05, ** *P* < 0.001, ***P < 0.0001

The number of living children affects women’s use of contraceptive at the individual level. Women with 1–4 children have higher odds of using contraceptive OR = 3.6(95% CI 2.8, 4.7) than women with no living children. Even the odds were highest in women having living children between five and eight 4.0(95% CI 2.8–5.5), although the odds have slightly decreased for women having more than 8 children there was a significant difference with those no living children at all 3.2(95% CI 1.7, 6.1). Wealth index was another factor influencing women’s use of contraceptive, as wealth index increases from poor to rich the odds of using contraceptive was also increased. Compared to the poor the middle and the rich had higher odds 1.4(95% CI 1.1, 1.7).

Among several husband’s characteristics that affect women’s use of contraceptive methods, one was partner’s occupation. Different occupations had different odds for women’s utilization, professional/technical/managerial and Clerical/sales/services/skilled labor positions of the husband gave highest of odds 1.9, while farming/unskilled followed with odds of 1.6. working women during the time of the survey had higher odds with 1.3(95% CI 1.1, 1.5), and experience of terminated pregnancy had increased the use of contraceptives in currently in-union women. Women’s individual level determinant factors were summarized in Table 3.

**Table:** 

Random effects	Model 0	Model I	Model II	Model III
ICC (%)	40.0%	23.0%	16.0%	14.0%
PCV	NA	82.5%	88%	90%
Model fitness				
Log likelihood	3927.16	− 3636.12	− 3634.10	− 3454.60
AIC	3858.32	7334.23	7304.17	7003.21
BIC	387.99	7545.98	7427.16	7324.25

### Community level determinants

To examine if the characteristics of the cluster affect women’s contraceptive use, regardless of women’s individual characteristics, we analyze community-level attributes. In Model II only community-level variables were added. Cluster characteristics like region, place of residence, religion, and community-level poverty were the factors associated with contraceptive use. The ICC in this model implied that difference between communities account for about 16% of the variation in women’s contraceptive use. The PCV also showed that 88% of the variation in contraceptive use between communities was explained by community level characteristics. As a cluster factor region was a strong predictor of contraceptive use. Considering Addis Ababa, the capital of the country as a reference, dwelling in Amhara region had the highest odds OR = 2.7 (95% CI 1.8–4.1) of contraceptive use. On the contrary Somali region had the lowest odds for contraceptive use OR = 0.1(95% CI 0.05, 0.2) compared with the people of the capital.

Compared with urban women, rural women had lower odds for contraceptive use OR = 0.7(95% CI 0.5,0.9), and community-level poverty decreased odds. Wealthy clusters had higher odds than poor clusters, regardless of household wealth index OR = 1.7(95% CI 1.4, 2.1). Religious affiliations had an effect on contraceptive utilization, being in a Muslim dominant cluster had lower odds of utilizations OR = 0.6(95% CI 0.5, 0.9) after controlling other individual and community level factors. Controlling other individual and community level factors, hearing information about family planning on radio and other information sources in the last few months didn’t contribute for utilization P value > 0.05 in all cases.

## Discussion

Family planning is one of the four pillars of safe motherhood initiative to reduce maternal death in developing countries. Contraceptives prevent unwanted and unplanned pregnancies, as well as complications, arising from these conditions. Increasing contraceptive use in developing countries has cut maternal death by 40% in past 20 years, a further 30% maternal deaths could be averted by fulfilling the unmet needs of family planning [21, 22]. Besides, there are non-contraceptive benefits of using contraception. Contraception can improve perinatal outcomes and child survival mainly by lengthening interpregnancy gaps as shortening the interpregnancy gap have a high risk of prematurity, low birth weight and infant death [23, 24].

In the current EDHS, about 34.9% of married women used modern contraceptive methods. This figure was much higher than previous percentages of EDHS data. In 2000’s and 2005’s DHS data contraceptive use was 8 and 14% respectively [25], further increment in contraceptive use was seen in 2011’s DHS survey around 27.3% [26]. Despite the unmet need for modern contraceptives, Ethiopia showed noteworthy improvement in the percentage of users from previous surveys. This percentage in this study is consistent with EDHS 2016 report of 35% [20]. The prevalence of contraceptive use in Ghana (2015) was 18.3% in adolescent females [27], in South Africa (2017) was 41.8% [28], and 32.1% in Uganda in 2011 [29]. There were changes annually in modern contraceptive prevalence rates in all women across sub-Saharan Africa with 1·92 percentage points (95% CI 1·14 to 2·70) generally [30].

Household wealth index positively affects contraceptive use in the current study, as found previously [31–33]. In Ethiopia modern contraceptives are available free of charge, the contribution of household wealth for contraceptive use will not be explained by the ability to pay for the service. Rather it reflects the general socioeconomic position of the household [34], which fundamentally affects health [35]. Economic status generally is known to increase not only contraceptive use but also other maternal health services in developing countries [36]. The two-way association of maternal health service utilization and contraceptive use have been explained previously [37]. The number of living children determines the odds of using contraception in this study, similar associations were found in the DHS of other countries [38–40]. Having living children between 1 and 4 had higher odds, and the odds increased in women having 5–8 children. In nulliparous women the desired number of children is unmet and the intension to bear a child is high and less likely to use contraceptives. As the number of children increased, women tend to use contraceptive as their desired number of children will be met. While great grand multiparous women might have had no idea about the use of modern contraceptives at all, or sex preference might be there in some women [41].

Women age had also effects on contraceptive use, where younger women had higher odds than older women in child bearing age. Although the denominators are different, similar scenarios have occurred in previous studies [42, 43]. There were improvements in female schooling in Ethiopia in the last few decades, younger females in this DHS might have better educational attainment. Women education at individual level was determinant in this study as well as in the previous studies [44, 45]. Schooling has positive relationship with contraceptive use and negative relationship with fertility [46], in Ethiopia there are evidences as well. Schooling might have decreased early age fertility and increased contraceptive utilization [47].

Husbands have a determinant role in contraceptive use of their wife, and approval by the husband gives the women higher chance of contraceptive use [48]. If husband’s occupation is professional/technical/manager, women have the higher odds of using contraceptive in this study. These husband’s occupation may reflect husbands educational status and contributed for contraceptive use by women as indicated [49]. There is a higher possibility that these women can reside in urban regions.

At the community level, there was lower odds of contraceptive use in Muslim dominant clusters. Similarly, women in Muslim communities of Asian countries usually had more children, desire for additional children and if they desire no more children, less likely to use contraceptive methods than the non-Muslims [50]. Urban women uses contraceptive more than the rural, this finding is in agreement with other studies conducted in Ethiopia and different parts of the world [51–53]. Women in the urban areas might have better decision-making confidence, autonomy, availability of contraceptive methods and even better living standards than the rural women. Rural women are prone to gender inequality and female reproductive decisions are dominated by males [54]. Longer distance to the health facility in rural areas was among the barriers [54], more employment opportunities are present in urban areas.

Community level women employment and community level poverty were also significant cluster characteristics affecting contraceptive use. These factors were significant in different studies [55–57]. Economically poorer areas have deficient health facility, even the distance to the health facility will be far. Poorer communities will not invest on women education and women empowerment will be less. Women in higher employed cluster tend to have assertiveness and decision-making power. Community cultural barriers might be minimal in these communities as well.

This study has some strengths**.** The study used the most recent and representative nationwide surveys. The study also, applied a multilevel analysis to accommodate the hierarchical nature of the Ethiopian Demographic Health Surveys data. Having the above strengths, the study has also some limitations: Since the data used in this study was cross-sectional data, which limits the conclusions about the causality of the factors on the dependent variable. Moreover, recall bias and not using sampling weight for the analysis were the limitations of the study.

## Conclusion

In nutshell, this study indicated both individual and community level factors can influence the use of contraception by women. Wealth index, women’s age, number of living children, husband’s occupation, ever experience of a terminated pregnancy, current working status of the women, number of births in the last 3 years, and hearing of FP messages through different media were significantly associated individual-level factors. Cluster characteristics like region, place of residence, religion, and community-level poverty were the factors associated with contraceptive use. Individual and community characteristics were significant predictors of use of modern contraceptives in Ethiopia and thus, these factors should be considered in programming for family planning in the country. Both individual and community-level characteristics should be priority areas in future measures to be taken in policies regarding contraceptive use in Ethiopia.

## Data Availability

The dataset supporting the conclusion of this article is available in the demographic health and survey repository in http://dhsprogram.com/data/.

## Abbreviations

CI: Confidence interval
EDHS: Ethiopian Demographic and Health Survey
FP: Family planning
HDI: Human development index
ICC: Intra class correlation
OR: Odds ratio
PCV: Proportional change in variance
